# Clinical characteristics and outcomes of adult patients admitted to acute care settings for alcohol withdrawal syndrome

**DOI:** 10.5339/qmj.2026.6

**Published:** 2026-03-05

**Authors:** Aram Salehi, Manish Barman, Memon Noor Illahi, Bassem Naif Altaleb Alhariri, Abdulqadir J Nashwan, Kalpana Singh

**Affiliations:** 1 Hazm Mebaireek General Hospital, Hamad Medical Corporation, Doha, Qatar; 2 Nursing & Midwifery Research Department, Hamad Medical Corporation, Doha, Qatar *Email: KSingh1@hamad.qa

**Keywords:** Alcohol withdrawal syndrome, clinical outcomes, benzodiazepines, Hospital Outcomes; Retrospective Cohort Study

## Abstract

**Background:**

Alcohol consumption is associated with significant morbidities such as liver disease, cardiovascular issues, and mental health disorders like depression and anxiety. Addressing acute alcohol withdrawal syndrome (AWS) effectively is essential to improving the health outcomes of individuals with alcohol use disorders. This study aims to evaluate AWS management practices and outcomes, with a focus on symptom severity, treatment protocols, and factors associated with prolonged or complicated hospital stays.

**Methods:**

This retrospective cohort study analyzed medical records of patients admitted to Hazm Mebaireek General Hospital (HMGH) with AWS between November 1, 2018, and October 31, 2021. Parameters assessed included demographic details, symptom severity using scales like Cushman’s and SOFA, benzodiazepine treatment protocols, and outcomes such as length of stay, ICU admissions, and mortality.

**Results:**

A total of 98 male patients were included, with a mean age of 39.9 ± 9.7 years. Among these, 51% were Indian nationals, followed by Nepalese (33%). Symptoms ranged from nausea (33%) to agitation (36%) and tremors (67%). Severity levels of AWS varied, with 59% experiencing mild symptoms, 34% moderate, and 7% severe. Fixed-dose and symptom-triggered benzodiazepine regimens were applied inconsistently, often deviating from recommended guidelines. Complications included seizures in 14% of cases and ICU admissions in 4%. The average hospital stay was 4.7 ± 5.1 days.

**Conclusion:**

AWS predominantly affects young males, with a significant burden on healthcare resources. Treatment strategies often diverged from international guidelines, emphasizing the need for standardized protocols to improve care quality and reduce variability in outcomes.

## 1. INTRODUCTION

Up to 40% of individuals report significant alcohol use or alcohol use disorder (AUD) during their lifetime, making alcohol a widely consumed psychoactive substance. Globally, up to 5.3% of deaths are attributable to AUD, which is associated with considerable morbidity and mortality.^1^ Harmful alcohol use poses diverse health risks and contributes to various morbidities, including liver dysfunction, mental health disorders, and physical injuries.^2^ Alcohol withdrawal syndrome (AWS) occurs in up to 50% of patients with AUD after a reduction or cessation of heavy, chronic drinking. AWS is characterized by symptoms such as tremors, hallucinations, anxiety, insomnia, and—in severe cases—seizures.^1^ Implementing a clinical pathway to identify AWS has been shown to improve patient outcomes and decrease hospital stays.^3^ The Clinical Institute Withdrawal Assessment for Alcohol (CIWA) scale remains the most widely used tool for diagnosing and monitoring AWS symptoms.^3^

Alcohol withdrawal symptoms generally emerge within 6 to 8 hours after a rapid decrease in blood alcohol levels, peak at around 72 hours, and typically subside within 5 to 7 days. In chronic alcohol users, glutamate neuromediation becomes upregulated to offset increased Gamma-aminobutyric acid (GABA ) activity from alcohol. When alcohol use decreases, there is initially a reduction in GABA activity, resulting in an imbalance with persistently high glutamate activity. This glutamatergic overactivity drives the symptoms of alcohol withdrawal. Each episode of AWS can harm brain and cognitive function, as glutamate is neurotoxic to the central nervous system.^4^

Currently, AWS treatment at Hazm Mebaireek General Hospital (HMGH) is based on the discretion of individual clinicians due to the absence of a standardized protocol. Existing guidelines recommend symptom-triggered long-acting benzodiazepines for mild-to-moderate cases and front-loading for severe symptoms, but inconsistent application highlights the need for local protocols to standardize care. There is growing international demand for information on alcohol use, related harm, and policy responses.^5^

Recent research highlights the hospital burden associated with AWS. Steel et al. reported significant age-, sex-, and race-based differences in the rates and proportions of AWS-related hospitalizations, with individuals under 60 years experiencing AWS hospitalizations at rates equal to or surpassing those for other chronic diseases. This underscores the importance of prioritizing identification, treatment, and monitoring of AUD within health systems.^6^ Laswi et al. found that elderly patients had longer hospital stays for alcohol withdrawal delirium and more frequent electrolyte imbalances than younger patients. Furthermore, middle-aged and older adults—particularly women—are experiencing increased rates of alcohol use and binge drinking, resulting in greater mortality, longer hospitalizations, and higher health care costs compared with youth.^7^

Abdelnaby et al. conducted a retrospective study in Qatar (2009–2012) and found that 38% of screened patients had positive blood alcohol concentrations; most were male (97%), with a mean age of 37.5 ± 11.6 years.^8,9^ Despite strict societal and legal restrictions in Qatar, alcohol dependence persists, especially among laborers. A systematic approach to diagnosing and managing AWS is critical to improving patient care in this region. To date, no study has focused on alcohol-related hospital admissions and management among laborers in Qatar. Establishing evidence-based care standards for alcohol addiction could encourage effective management pathways for this population. This study seeks to develop a clinical pathway tailored specifically for laborers in Qatar.

## 2. METHODS

### 2.1 Study design and setting

This retrospective observational study was conducted at Hamad Medical General Hospital (HMGH). We reviewed medical records of adult patients admitted for AWS between November 1, 2018, and October 31, 2021.

### 2.2 Eligibility criteria

#### 2.2.1 Inclusion criteria

- Adults with a clinical diagnosis of AWS per standardized criteria (CIWA-Ar score ≥8), assessed by the admitting physician.
- Received thiamine during hospitalization.

#### 2.2.2 Exclusion criteria

- Last alcohol consumption less than six hours before admission.
- Daily use of AWS treatment medications (e.g., benzodiazepines, barbiturates, or clomethiazole) in the previous 30 days.
- Major cognitive, psychiatric, or medical comorbidity.
- Opiate or stimulant dependence.

### 2.3 Diagnostic and assessment protocol

Diagnosis of AWS was based on the patient’s clinical history, physical examination, and CIWA-Ar score. The necessity of thiamine administration was determined to prevent Wernicke’s Encephalopathy. Relevant clinical documentation—including AWS symptom severity, alcohol consumption patterns, and management steps—was systematically reviewed by a team of clinicians familiar with established AWS treatment guidelines.

### 2.4 Data collection procedures

A team of trained clinicians independently extracted data using a standardized data collection form. The following parameters were recorded:

- Patient demographics (age, sex, nationality).
- History of alcohol consumption.
- Documented AWS symptoms and CIWA-Ar scores.
- Benzodiazepine dosage and administration patterns.
- Thiamine administration.
- Laboratory findings.
- Involvement of psychiatry and other consultation teams.

Discrepancies in data abstraction were resolved by consensus, and inter-rater reliability was assessed to ensure data consistency.

### 2.5 Outcomes

#### 2.5.1 Primary outcome

- Variation in clinical practice regarding the correct use of the CIWA-Ar scale and adherence to international AWS management guidelines at HMGH.

#### 2.5.2 Secondary outcome

**Figure 1.**
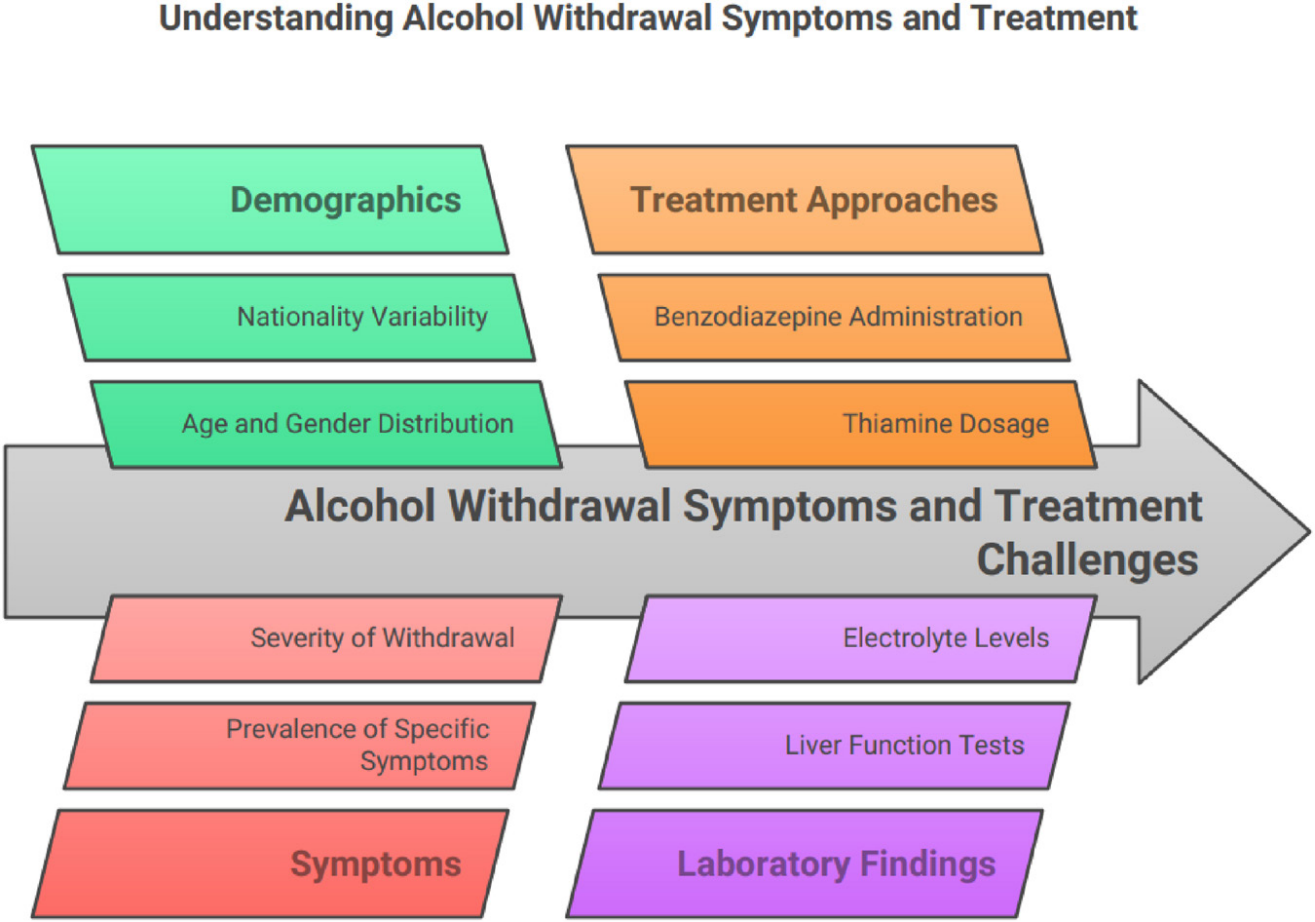
Challenges facing the clinicians in treating Alcohol Withdrawal Syndrome.

- Improvements in patient care linked to the development and implementation of local AWS management guidelines (Figure 1).

### 2.6 Data management and statistical analysis

Descriptive statistical analyses were conducted to summarize the demographic, clinical, and laboratory characteristics of the study participants. Categorical variables such as nationality, presence of alcohol withdrawal symptoms, comorbidities, types of benzodiazepine and thiamine treatments, and psychiatric consultation outcomes were summarized using frequencies and percentages. Continuous variables, including age, vital signs, severity scores, duration of treatment, laboratory values, and length of hospital stay, were summarized using means and standard deviations (mean ± SD). As this study was primarily descriptive in nature, no inferential statistical tests were performed, and therefore p-values and confidence intervals were not calculated. All statistical analyses were performed using STATA 17.0, and results were presented in tabular and graphical formats to illustrate key findings.

## 3. RESULTS

### 3.1 Demographics

**Figure 2.**
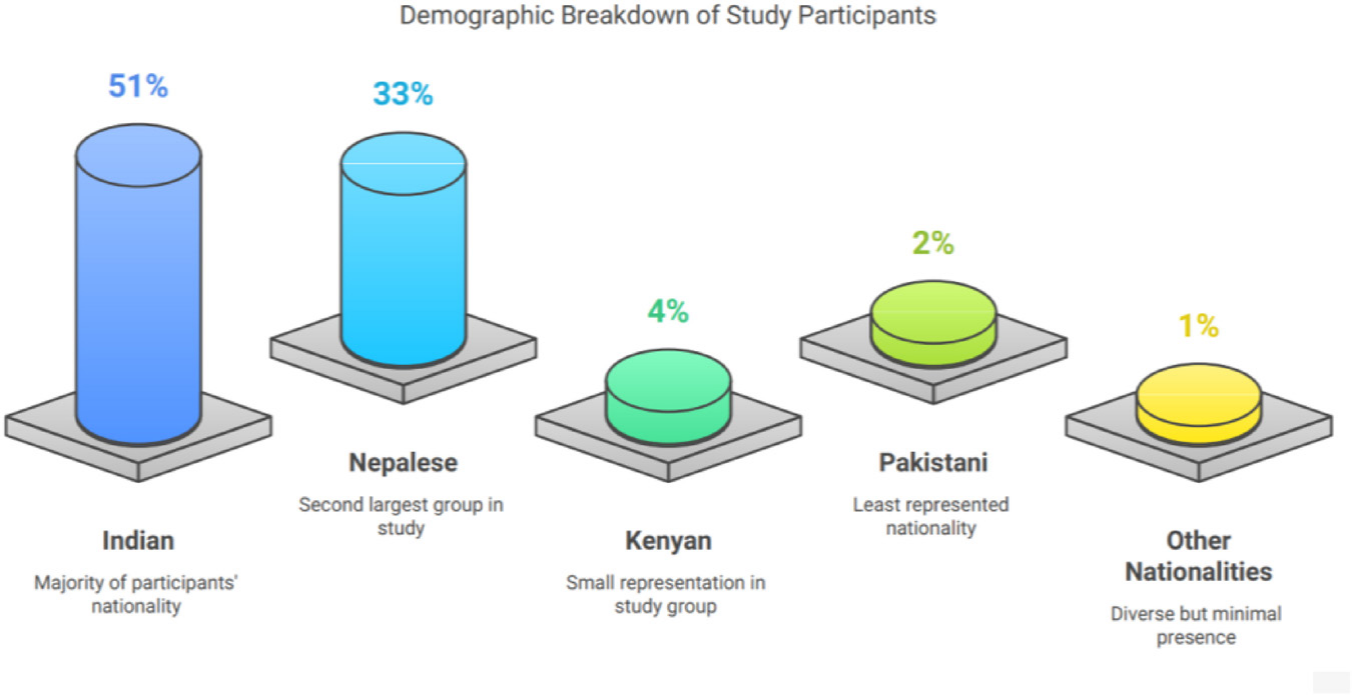
Nationality distribution of study participants.

A total of 98 adult patients with a clinical diagnosis of AWS were included in the study. The cohort was predominantly male (100%), with a mean age of 39.9 ± 9.7 years. Most patients were Indian nationals (51%), followed by Nepalese (33%), with other nationalities including Kenyan (4%), Pakistani (2%), and others comprising the remaining 10% (Table 1; Figure 2).

### 3.2 Symptom distribution

**Figure 3.**
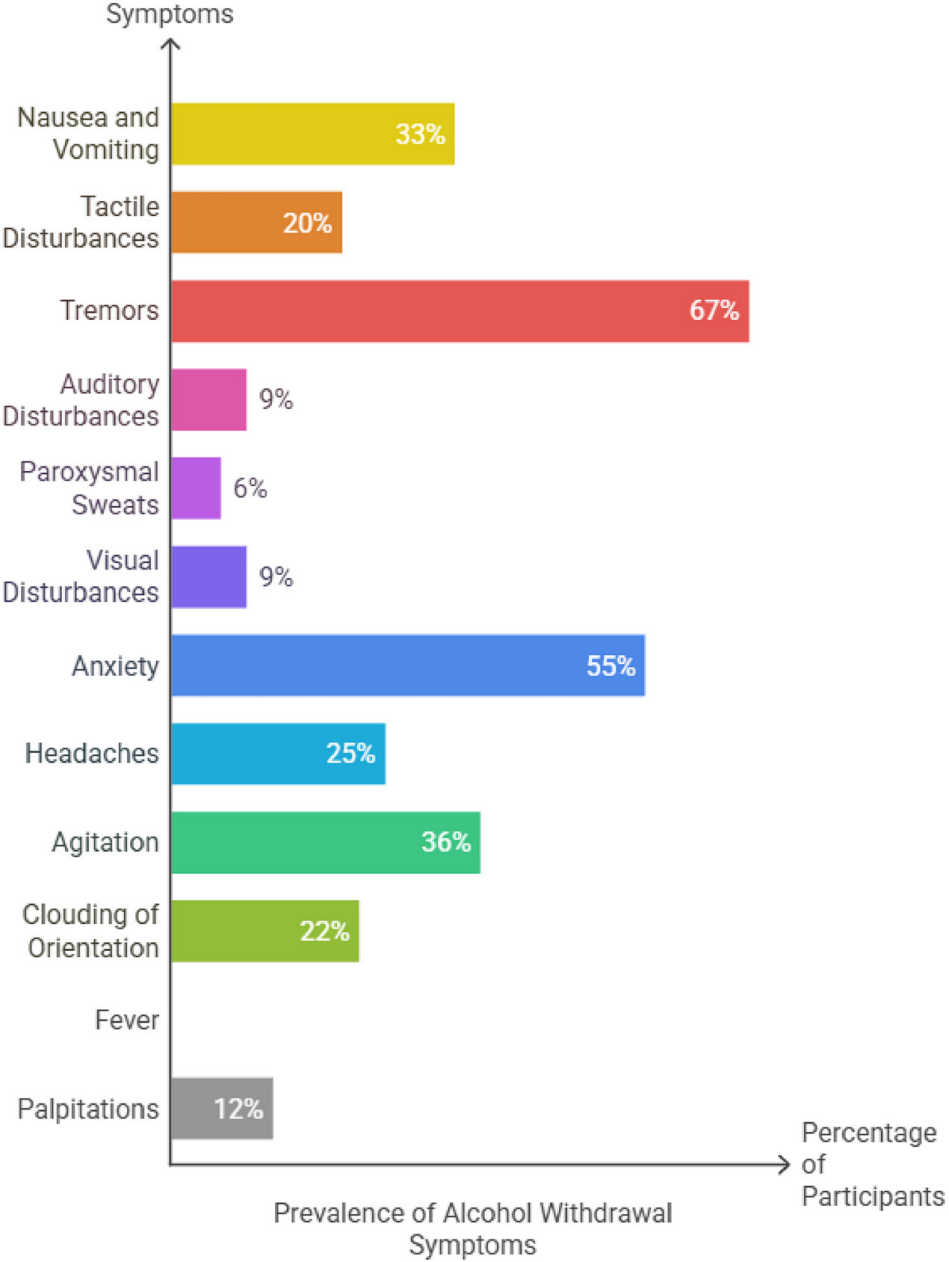
Percentage of alcohol withdrawal symptoms.

Symptoms varied in prevalence: tremors were reported by 67% of patients, nausea/vomiting in 33%, agitation in 36%, and anxiety in 55%. Visual or auditory disturbances were less common, observed in roughly 9% of cases. Clouding of orientation occurred in 22%, while 10% had fever (Table 2; Figure 3). Regarding severity, 59% had mild AWS, 34% moderate, and 7% severe based on clinical assessment. Notably, 14% experienced seizures, and 4% required ICU admission.

### 3.3 Alcohol use and hospitalization history

**Figure 4.**
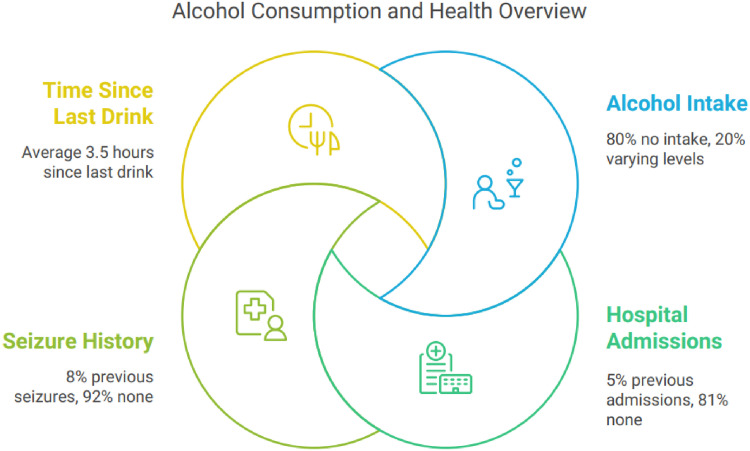
Alcohol consumption and health status.

Despite AWS diagnosis, 80% of the patients denied recent alcohol intake at admission, likely due to underreporting linked to stigma. The mean time from last drink to admission was 3.5 ± 4.7 hours. Previous alcohol-related admissions were reported in 13% of patients, while 8% had a history of alcohol-related seizures (Table 3; Figure 4).

**Figure 5.**
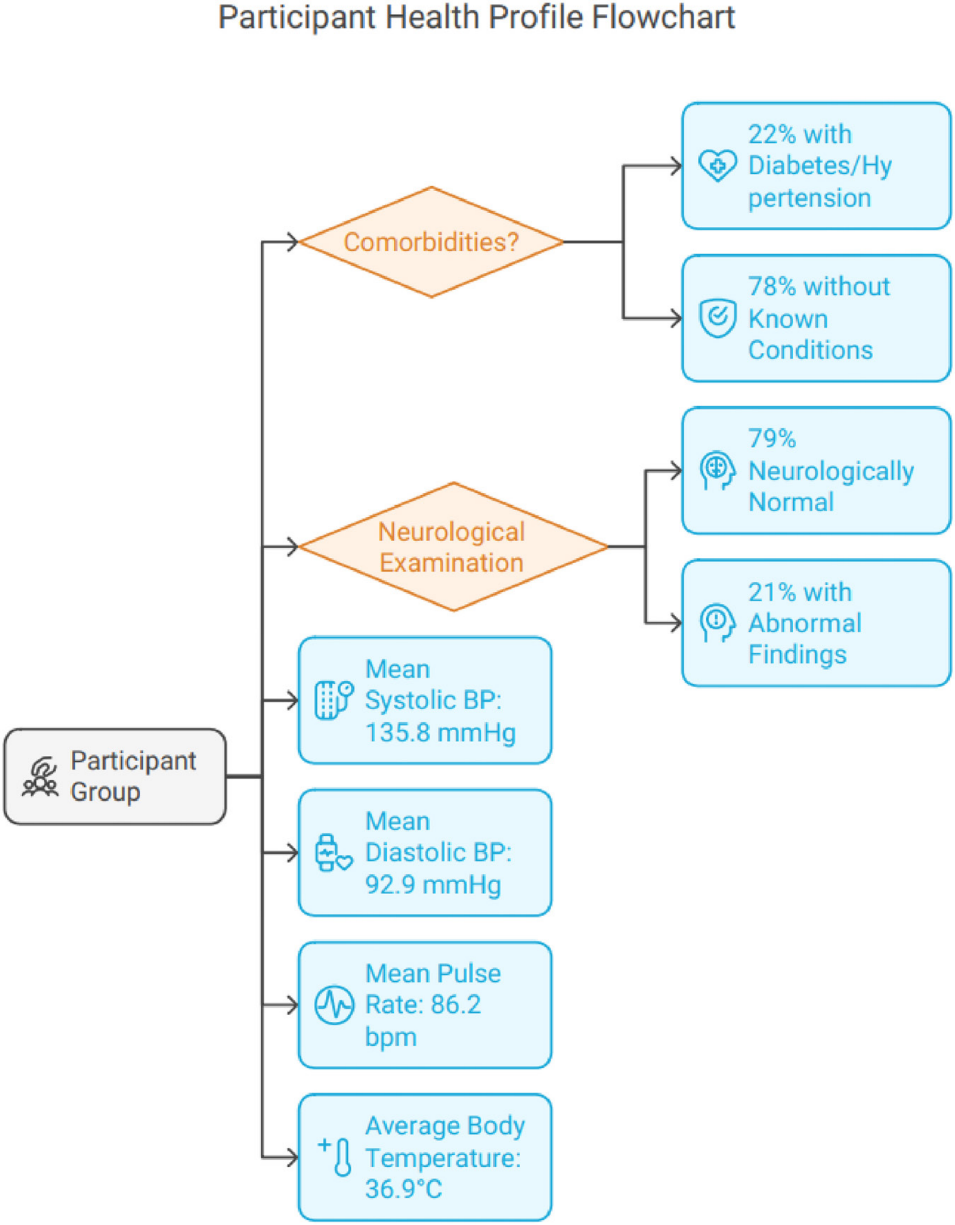
Health profile of study participants.

In terms of comorbidities, 22% of participants had a past medical history of conditions like diabetes or hypertension, while 78% had no known medical history. Neurological examinations revealed that 79% of participants were neurologically normal, while 21% had abnormal findings. The mean systolic blood pressure (BP) was 135.8 ± 18.5 mmHg, and the diastolic BP was 92.9 ± 59.8 mmHg. The mean pulse rate was 86.2 ± 15.4 beats per minute, and the average body temperature was 36.9 ± 0.6°C (Figure 5).

### 3.4 Vital signs

Initial vital signs reflected typical AWS autonomic responses: mean temperature was 36.9 ± 0.6°C, pulse rate 102 bpm, and BP roughly 135/80 mmhg.

Vital signs were reassessed 24 hours after admission. The mean systolic BP had decreased to 126.7 ± 12.6 mmHg, and the diastolic BP dropped to 80.9 ± 11.3 mmHg. The average pulse rate had improved to 78.8 ± 10.9 beats per minute, while the body temperature remained stable at 36.9 ± 2.8°C. Neurological complications such as nystagmus were absent in all patients (100%), while ophthalmoplegia was noted in 1% of participants.

**Figure 6.**
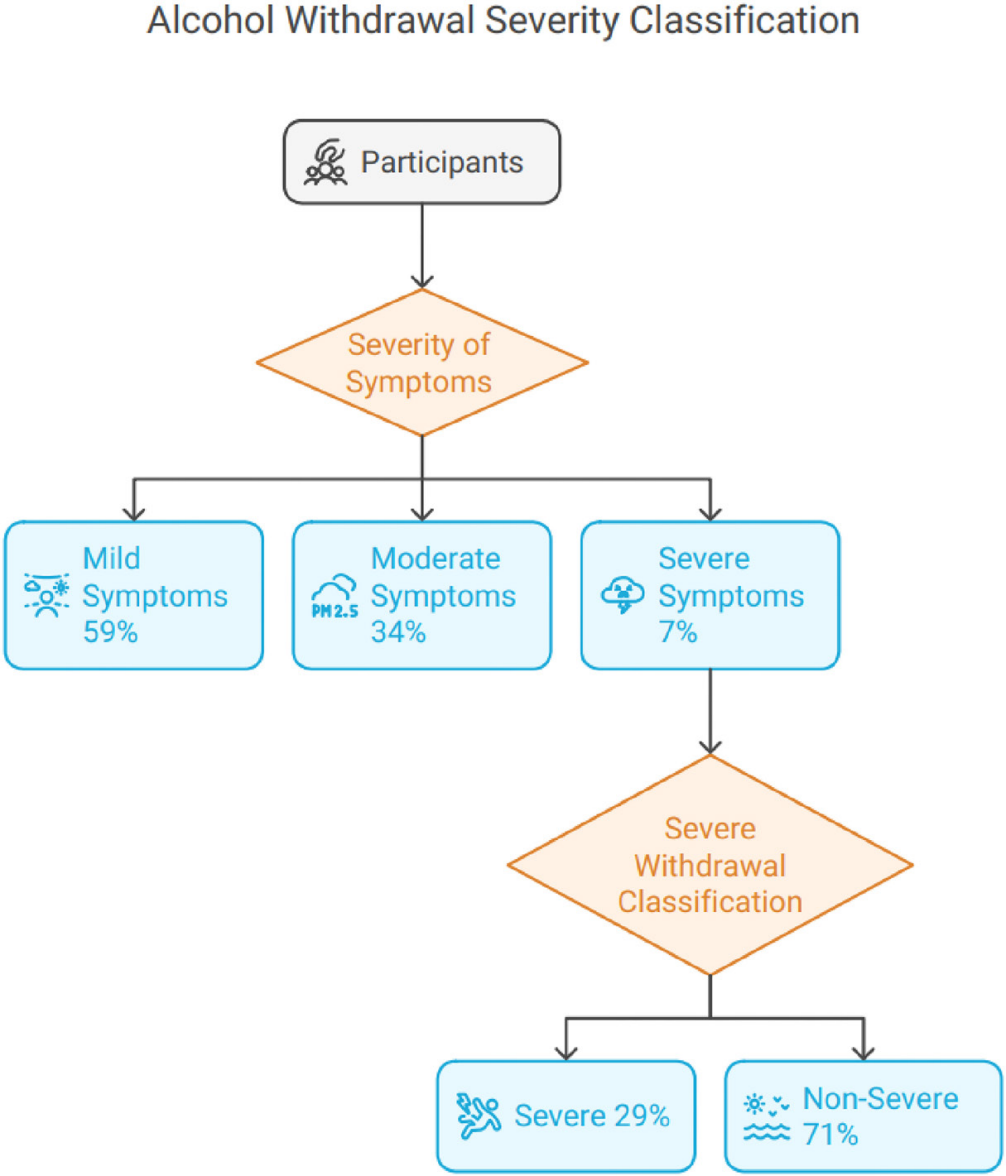
Alcohol withdrawal severity classification.

The severity of alcohol withdrawal varied. Mild symptoms were reported by 59% of participants, moderate symptoms by 34%, and severe symptoms by 7%. The severity scale score (C/J) averaged 4.4 ± 4.1. In terms of severe withdrawal, 29% of participants were classified as having severe symptoms, while 71% were categorized as non-severe (Figure 6).

### 3.5 Laboratory findings

**Figure 7.**
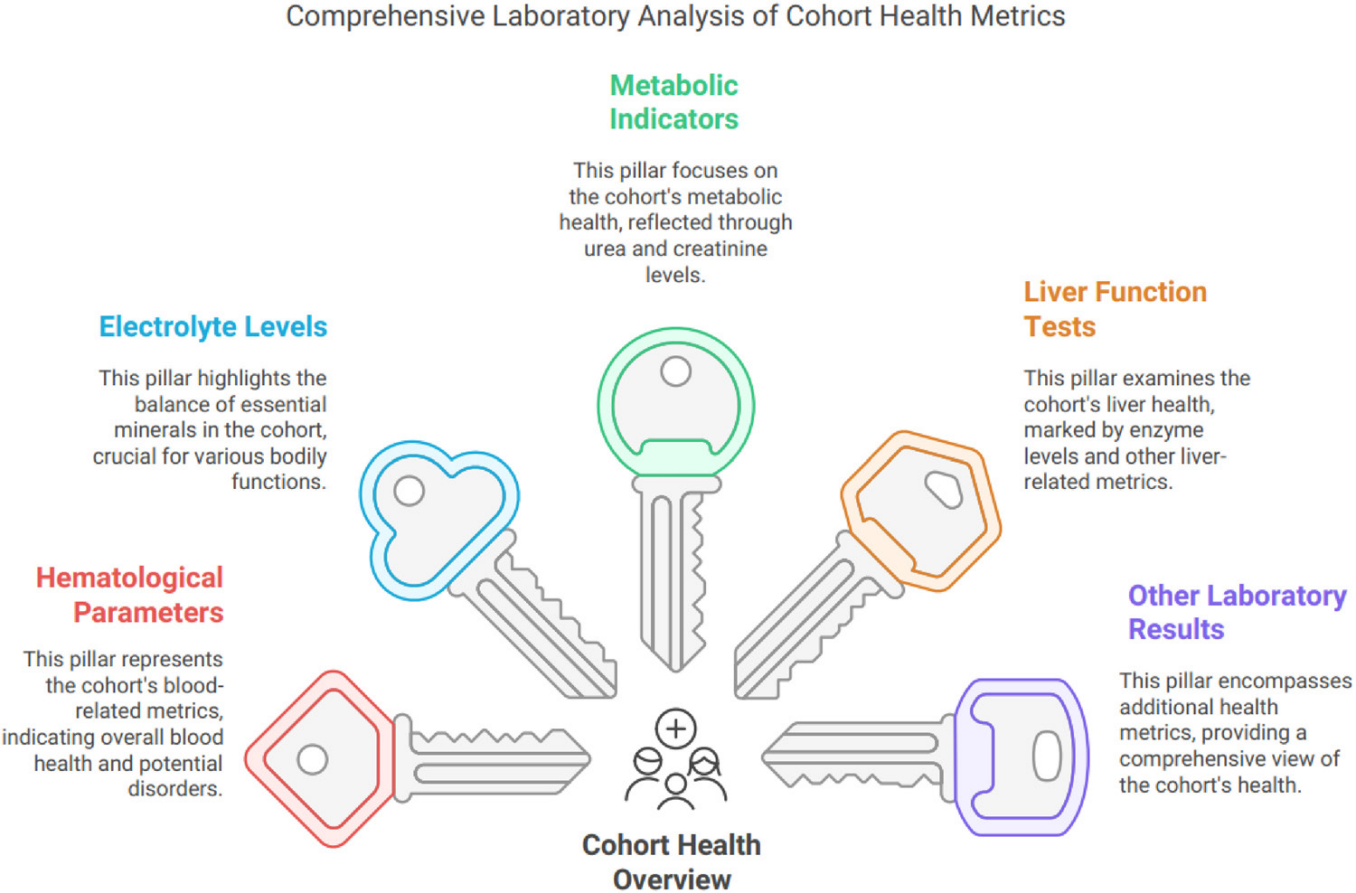
Cohort health metrics with laboratory results.

Electrolyte disturbances were common: hyponatremia was documented in 18%, hypokalemia in 12%, among others. Elevated liver enzymes were present in 35.7% of patients, indicating hepatic stress. Blood alcohol was positive on admission in 20% to 22% of those tested (Table 4). Other labs, such as hemoglobin, Mean Corpuscular Volume (MCV), glucose, and magnesium, were within expected ranges as per (Table 4; Figure 7).

### 3.6 Treatment details

**Figure 8.**
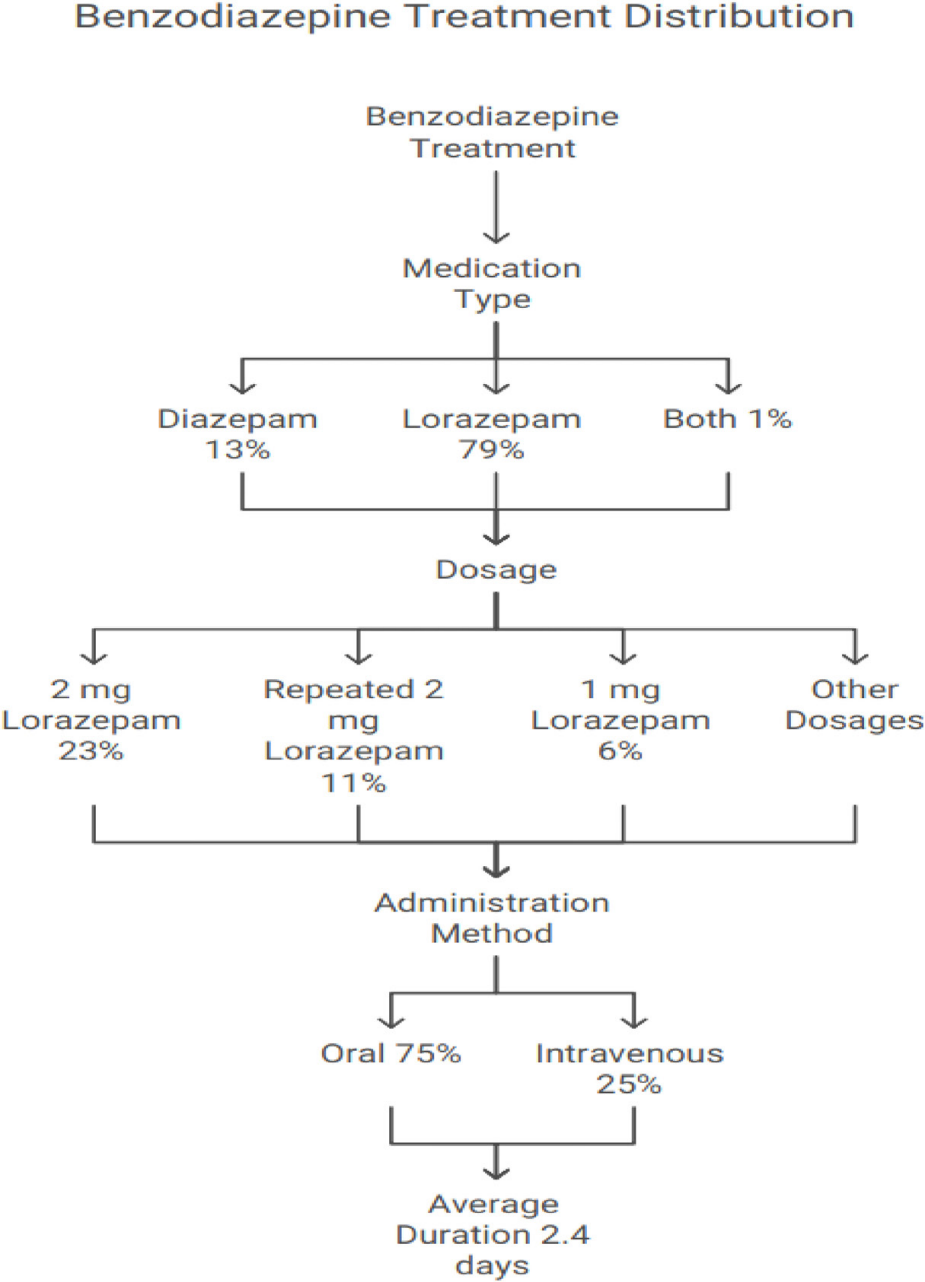
Overview of benzodiazepine treatment.

Benzodiazepines were the mainstay of treatment, with lorazepam being the most commonly used agent (79%), followed by diazepam alone (13%). Different dosing regimens were observed, predominantly symptom-triggered rather than fixed dosing. The average benzodiazepine dose was 1 to 2 mg lorazepam daily or equivalent, administered mostly intravenously (88%). Thiamine was uniformly administered to all patients, predominantly IV once daily, for an average of 3.6 ± 2.5 days (Table 5; Figure 8).

**Figure 9.**
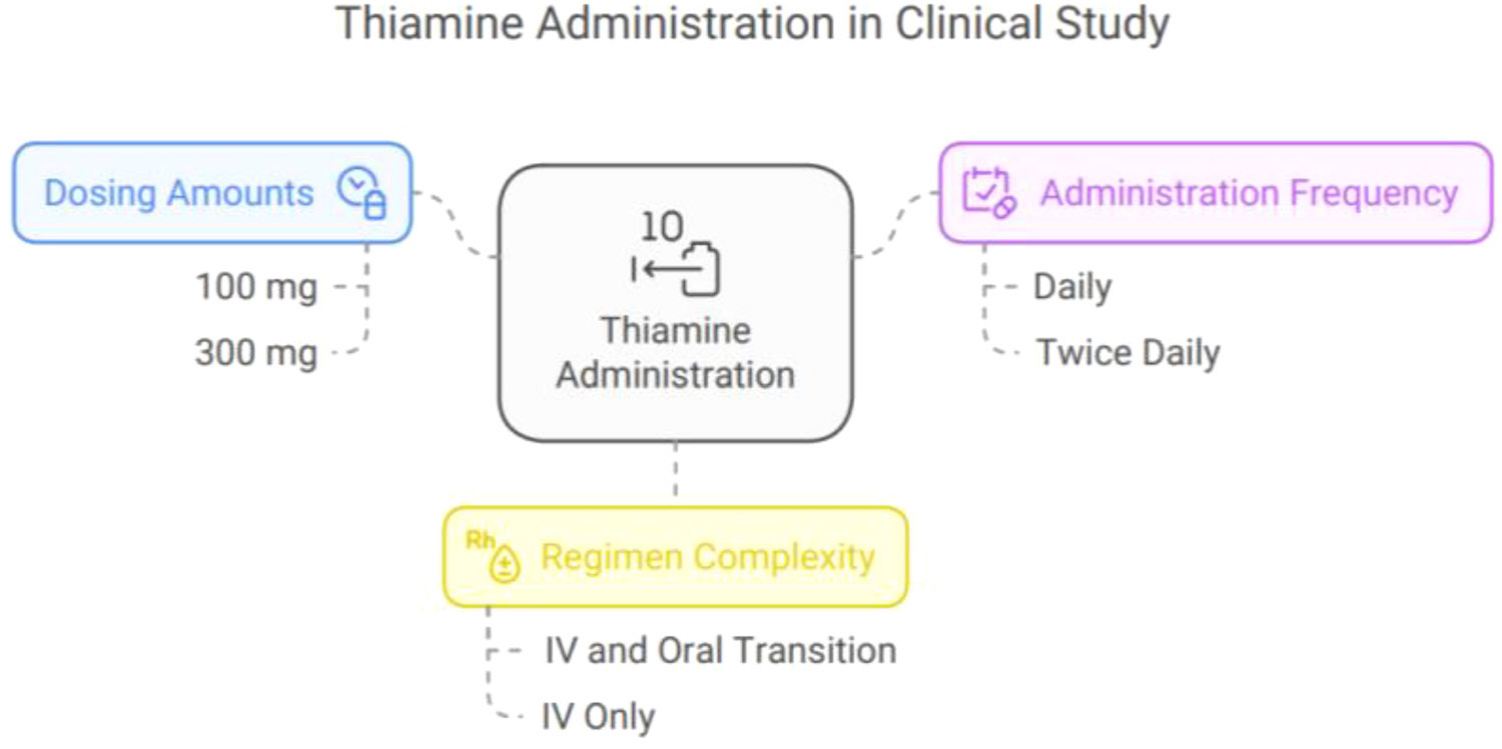
Overview of thiamine treatment.

Thiamine was administered for most patients, with 82.6% receiving 100 mg and 20% receiving 300 mg. Some participants had more complex regimens, such as 1% who received 300 mg IV for 2 days followed by 100 mg IV, then transitioned to oral dosing. Another 1% received 300 mg IV for 2 days, followed by 100 mg orally for an additional 2 days. Thiamine was administered daily in 96% of patients, while 4% received it twice daily. The average duration of thiamine administration was 3.6 ± 2.5 days (Figure 9).

### 3.7 Psychiatric evaluation

Psychiatric consultation was obtained in 45% of patients to assist with withdrawal management and psychosocial needs. About 55% had no documented psychiatric evaluation (Table 6).

### 3.8 Clinical outcomes

**Figure 10.**
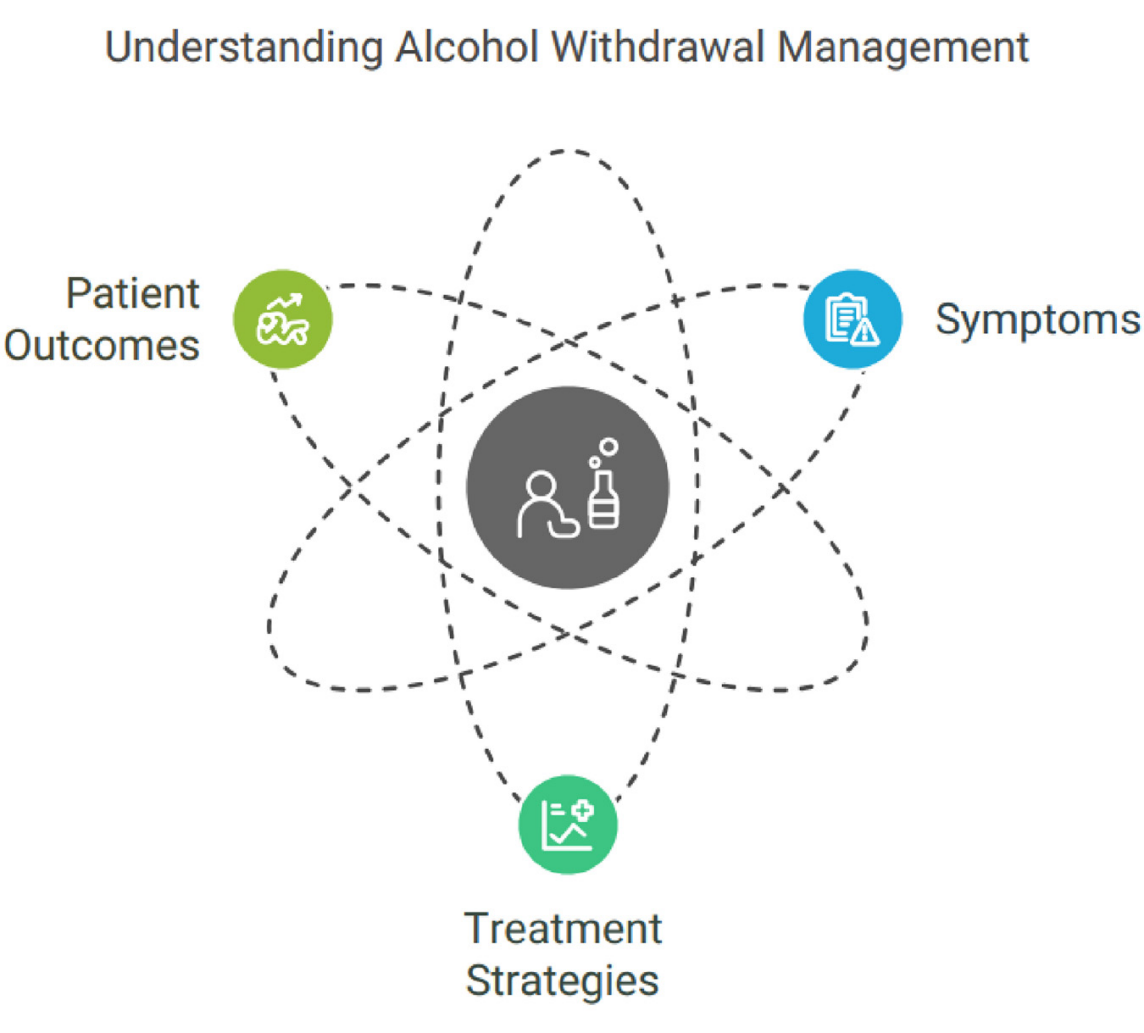
Understanding the alcohol withdrawal management.

Mean hospital stay was 4.7 ± 5.1 days. Severe AWS cases had longer hospitalization compared to mild/moderate cases (data for detailed comparison could be added if available). ICU admission was required in 4% of cases, mostly due to severe withdrawal complications such as seizures or delirium tremens. No in-hospital mortality was reported (Table 7; Figure 10).

## 4. DISCUSSION

This study presents a comprehensive evaluation of adult patients hospitalized for AWS at a general hospital in Qatar, with a predominant focus on the expatriate workforce. Our findings reveal high prevalence rates of classical AWS symptoms—including tremors, agitation, and anxiety—alongside significant laboratory derangements, such as electrolyte imbalances and elevated liver enzymes. These results align with existing literature indicating that complicated AWS is frequently associated with underlying liver dysfunction, macrocytosis, and metabolic disturbances, especially in populations with sustained high-risk alcohol consumption.

Legal and cultural constraints surrounding alcohol use in Qatar created substantial barriers to both disclosure and care. Most patients denied recent alcohol intake, despite clear clinical and laboratory evidence of withdrawal. Such underreporting, driven by stigma or fear of legal reprisal, likely limited accurate history-taking and may have influenced the assessment of AWS severity. Furthermore, the all-male sample is a notable limitation: while men are more often exposed to alcohol given the expatriate labor demographics, women with AUD may be under-identified and under-treated—an issue requiring further study. This single-gender representation limits the generalizability of our findings.

Another area warranting discussion is the relationship between AWS symptoms and vital signs. As elsewhere reported, mean pulse rates were elevated, and low-grade fever was frequent, reflecting the sympathetic overactivity inherent to acute withdrawal. However, a more detailed correlation analysis between specific symptom clusters and derangements in vital parameters (e.g., hypertension, tachycardia) was not conducted in our review and should be prioritized in future research to tailor supportive care approaches.

A critical practice concern is the observed lack of systematic correlation between benzodiazepine dosing and documented symptom severity. Most patients received a mixture of fixed and symptom-triggered regimens, and dosing decisions appeared driven more by provider preference than by guideline-based symptom scoring, particularly in the absence of a validated instrument such as the CIWA-Ar scale. Evidence suggests that symptom-triggered regimens, when guided by CIWA-Ar, can reduce benzodiazepine exposure and shorten hospital stays without increasing complications. Fixed-dose protocols are sometimes favored in resource-limited settings or where staff are less familiar with CIWA-Ar, and may be safer for patients with poor self-report or severe comorbidities. However, observational data—such as ours—do not permit conclusions about the comparative effectiveness of these approaches. Further, fixed-dose regimens have been associated with both under- and over-sedation and may lead to excess medication exposure in mild cases. Thus, our observation of inconsistent dosing without standardized assessment represents an important area for immediate quality improvement. The moderate rate of ICU admissions (4%) and high seizure complication rate (14%) may, in part, reflect gaps in individualized titration of sedation.^10–12^

The administration of thiamine to all patients represents a strength of our AWS care pathway, consistent with international guidelines aiming to prevent Wernicke’s encephalopathy—a well-recognized risk in this population. However, our detection of persistent laboratory abnormalities highlights ongoing gaps in pre-hospital and post-acute care: many patients demonstrated signs of chronic malnutrition or liver disease upon admission, yet discharge planning rarely included structured rehabilitation, nutritional support, or long-term follow-up. This is a critical area, as international studies have demonstrated that comprehensive, multidisciplinary continuity of care reduces both relapse and long-term morbidity following AWS admissions.^13–17^

### 4.1 Gap in psychiatric input: Interpretation and recommendations

Our data indicate that 55% of patients hospitalized for AWS did not receive psychiatric input during admission. Several factors may contribute to this gap:

- **System barriers:** Limited psychiatric staffing or prioritization of psychiatry referrals primarily for overt agitation, psychosis, or suicidal ideation rather than for all patients with AWS, despite evidence showing broader benefits from multidisciplinary input.
- **Provider practice patterns:** Medical teams may prioritize physical stabilization and acute withdrawal management, underestimating the importance of early psychiatric or addiction medicine consultation for long-term relapse prevention, risk assessment, and psychosocial care.
- **Cultural and legal context:** In Qatar, strong social stigma and legal restrictions surrounding alcohol may discourage patients from disclosing psychiatric symptoms or substance use histories, potentially reducing both appropriate referrals and patient engagement with mental health support.
- **Documentation issues:** In some instances, psychiatry may have provided informal advice, or patients may have been evaluated after discharge from acute care, neither of which was systematically recorded in the retrospective chart review.

Despite these challenges, the study demonstrates several strengths: robust thiamine delivery, recognition of post-acute care gaps, and an explicit focus on the intersection of AWS management with local socio-cultural factors. However, the predominantly descriptive design and lack of systematic AWS severity scoring are important limitations.

## 5. Conclusion

In summary, this study highlights the burden of AWS among predominantly male expatriate workers in Qatar and reveals both strengths and gaps in the current management pathway. Key priorities for improvement include:

Standardizing AWS protocols to incorporate validated symptom assessment tools (e.g., CIWA-Ar), enabling more individualized and evidence-based benzodiazepine administration.

Qualifying the use of fixed-dose benzodiazepine regimens, as benefits observed in this cohort may not translate broadly and are based on non-randomized data.

Explicitly acknowledging the gender limitation and the likely under-detection and under-treatment of AWS in women.

Enhancing post-discharge follow-up and access to multidisciplinary aftercare, including rehabilitation, to address chronic risks.

Institute targeted training for clinical staff around both the diagnosis and social/cultural determinants of AWS presentation in the local context.

### 5.1 Addressing this gap

To improve the rate and quality of psychiatric input, we recommend:

Automatic psychiatric referral triggers for all AWS admissions, or at a minimum for those with moderate/severe withdrawal, previous admissions, suicidality, or comorbidities.

Staff training to reinforce the holistic benefits of psychiatric involvement in AWS, extending beyond acute symptom management (including relapse prevention, family/social assessment, and motivational interviewing).

Improved documentation protocols to ensure all assessments and interventions are recorded, which can inform care transitions and multidisciplinary planning.

At a policy level, expanding awareness, improving training, and integrating rehabilitation services will be critical for reducing AWS morbidity and advancing quality of AUD care in settings like Qatar. Future research should address the effectiveness of protocolized, symptom-driven care in diverse patient populations and the impact of sociocultural barriers on both admission and recovery trajectories.

## ACKNOWLEDGEMENT

The content of this work is solely the responsibility of the authors and does not necessarily represent the official views of the Hamad Medical Corporation. All authors had access to the data supporting this study and a role in writing the submitted manuscript. The authors would like to thank Dr. Abdelkarim Hashim Mohamed, Dr. Abdalrahman Mohammed Mostafa, Dr. Reynaldo Jr. Balintona, Dr. Usamah Saad Mohammad Al Anbagi and Dr Ahmad Eid Nazzal Alharafsheh for their valuable contribution in data gathering.

## DISCLOSURE

The case manuscript, patient information, clinical data, and imaging are in accordance with international Institutional Review Board standards and Hamad Medical Corporation policy.

## CONFLICT OF INTEREST

The authors declare that there is no conflict of interest.

## ETHICAL APPROVAL

The study was approved by the ethical committee of the Medical Research Center at Hamad Medical Corporation, Doha, Qatar (approval number: MRC-01-22-206). The consent was waived due to the retrospective nature of the review. The confidentiality of the included subjects was maintained by not disclosing the identification details and using anonymized data. The study was conducted in full compliance with the principles of the “Declaration of Helsinki” and Good Clinical Practice (GCP).

## AUTHOR CONTRIBUTIONS

AS: study design, data collection, manuscript writing, editing. MB: study design, data collection, manuscript writing, editing. BAH: data collection, analysis, manuscript writing. MNI: conceptualization, data collection, manuscript editing. AN: study design, critical review, manuscript editing. KS: statistical analysis, writing the manuscript, editing, and critical review.

## Figures and Tables

**Table 1.** Demographic characteristics of patients with AWS (*n* = 98).

Characteristic	N (%)
Male	98 (100)
Age, mean ± SD (years)	39.9 ± 9.7
Nationality	
Indian	50 (51)
Nepalese	32 (33)
Kenyan	4 (4)
Pakistani	2 (2)
Others*	10 (10)

*Includes British, Filipino, Jordanian, Moroccan, Qatari, Russian, Sri Lankan, and Swedish.

**Table 2.** Clinical symptoms in AWS patients (*n* = 98).

Symptom	Yes, N (%)	No, N (%)
Tremors	65 (67)	32 (33)
Nausea/vomiting	32 (33)	66 (67)
Agitation	35 (36)	62 (64)
Anxiety	53 (55)	44 (45)
Visual disturbance	9 (9)	88 (91)
Auditory disturbance	9 (9)	88 (91)
Clouding of orientation	21 (22)	75 (78)
Seizures	14 (14)	84 (86)
Fever	10 (10)	87 (90)

**Table 3.** Alcohol use and hospitalization history.

Parameter	N (%) or mean ± SD
Denied recent alcohol intake	78 (80)
Mean time from last drink (hours)	3.5 ± 4.7
Previous alcohol-related admission	13 (13)
Previous alcohol-related seizure	8 (8)

**Table 4.** Laboratory parameters.

Parameter	N (%) with abnormality or mean ± SD
Hyponatremia	18 (18)
Hypokalemia	12 (12)
Elevated liver enzymes	35 (36)
Blood alcohol positive on admission	22 (22)
Hemoglobin (g/dL)	14.3 ± 1.7
MCV (fL)	87.5 ± 6.0
Glucose (mmol/L)	6.7 ± 3.1
Magnesium (mmol/L)	0.8 ± 0.1

**Table 5.** Treatment details.

Treatment parameter	N (%) or mean ± SD
Lorazepam use	77 (79)
Diazepam use	13 (13)
Benzodiazepine administration route	IV 88 (88), Oral 10 (10)
Thiamine administration	98 (100)
Thiamine duration (days)	3.6 ± 2.5

**Table 6.** Psychiatric consultations.

Psychiatric evaluation	N (%)
Seen or evaluated	44 (45)
Not evaluated	54 (55)

**Table 7.** Clinical outcomes.

Outcome	Value
Mean length of stay (days)	4.7 ± 5.1
ICU admission	4 (4)
In-hospital mortality	0 (0)
